# Hepatitis Action Plan and Changing Trend of Liver Disease in Japan: Viral Hepatitis and Nonalcoholic Fatty Liver Disease

**DOI:** 10.5005/jp-journals-10018-1213

**Published:** 2017-05-05

**Authors:** Tatsuya Kanto, Sachiyo Yoshio

**Affiliations:** 1 The Research Center for Hepatitis and Immunology, National Center for Global Health and Medicine, Chiba, Japan

**Keywords:** Basic act on hepatitis measures, Basic guidelines for promotion of control measures for hepatitis, Interleukin 34, Nonalcoholic fatty liver disease.

## Abstract

In Japan, the estimated number of chronic hepatitis B virus infections was 1.1 to 1.4 million, and that of chronic hepatitis C virus was 1.9 to 2.3 million in 2000. The mortality of hepatocellular carcinoma had been increasing and hit the peak at around 2002, which subsequently started to decrease. Japan has a national action plan for addressing viral hepatitis called Basic Act on Hepatitis Measures, established in 2009. In 2011, basic guidelines for promotion of control measures for hepatitis were issued, comprising nine principles in order to promote measures to prevent hepatitis B and C. According to these guidelines, national and local governments share screening costs for testing hepatitis B and C in residents who are over 40 years old. Thus, out-of-pocket expenses from examinees are nil or reduced to the minimum. In addition, for patients with chronic hepatitis B or C and on treatment, drug prices of nucleotide analogs, interferon (IFN) treatment, or IFN-free direct antiviral agents along with examination expenses should be covered by special programs for viral hepatitis. The national and local governments cover the amount in excess of 100 to 200 USD of the cost of treatment. The proportion of liver cancer with nonviral etiology has been increasing in Japan. For the screening and follow-up of patients with nonalcoholic fatty liver disease, we demonstrated that interleukin 34 is a feasible fibrosis marker. Several advantages have prevailed in the Japanese health care systems for patients with viral liver disease compared with those in countries in the Western Pacific region. Therefore, Japan should take a lead in helping the implementation of practical hepatitis action plans in every country when in need.

**How to cite this article:** Kanto T, Yoshio S. Hepatitis Action Plan and Changing Trend of Liver Disease in Japan: Viral Hepatitis and Nonalcoholic Fatty Liver Disease. Euroasian J Hepato-Gastroenterol 2017;7(1):60-64.

## INTRODUCTION

Around the globe, approximately 350 million people are infected with hepatitis B virus (HBV) and 170 million are with hepatitis C virus (HCV) respectively. Both viruses are hepatotropic and principally noncytopathic in nature; the majority of the endemic areas are the developing countries. Once infected, substantial populations seamlessly progress to chronically infected states that eventually develop liver cirrhosis and hepatocellular carcinoma (HCC) in decades. Liver cirrhosis and liver cancer are responsible for 94% of deaths associated with hepatitis infections. Liver cancer is the second most common cause of cancer deaths in the Asia-Pacific region, and approximately 78% of liver cancer causes are a result of chronic viral hepatitis B or C.^1^ Viral hepatitis is the seventh leading cause of mortality globally, responsible for 1.45 million deaths in 2013.^2^ One quarter of the world’s population lives in the Western Pacific, but the region bears 40% of the world’s deaths caused by hepatitis. Consequently, hepatitis kills more than 1,500 people every day in the region.^1^

Regardless of the success of HB vaccination, which has reduced the prevalence of hepatitis B surface antigen (HBsAg) in the under 5-year-old population in several countries, millions of people across the region still continue to live with chronic hepatitis B or C and the risk of cirrhosis and liver cancer. We now have effective medicines, such as direct acting antivirals (DAAs) to manage and treat chronic hepatitis C. However, the high prices of such medicines are a major barrier for access to treatment across the region. We need innovative approaches to ensure that the people of the low- or middle-income countries can benefit from these life-saving medicines.

## NATIONAL HEPATITIS ACTION PLAN IN JAPAN

In 2000, the estimated number of individuals with chronic HBV infection was 1.1 to 1.4 million, and that of chronic HCV infection was 1.9 to 2.3 million in Japan. Since the start of the national surveillance, the mortality of HCC has been increasing, showing the peak at around 2002. Subsequently, such mortality turned out to be decreasing regardless of gender. Annual incidence of deaths in 2014 by liver cirrhosis was approximately 10,000 and that by liver cancer was 29,000. Also, 70% of deaths by liver cirrhosis or cancer were caused by hepatitis B and C. Therefore, in Japan, it has been an important health issue for the management and care of patients with chronic hepatitis B or C infection.

**Fig. 1:**
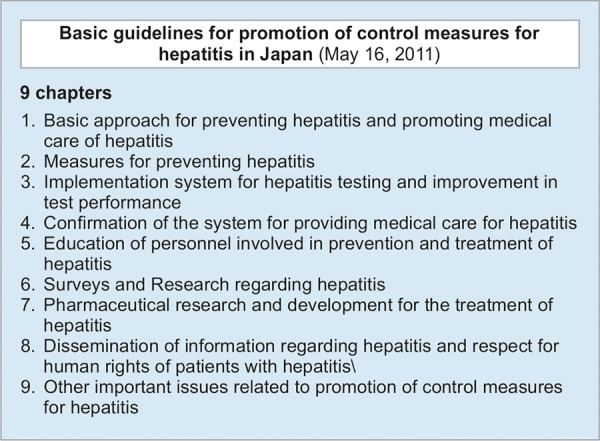
The Basic Act on Hepatitis Measures was issued in December 2009. In May 2011, basic guidelines for promotion on control measures for hepatitis were issued by the Ministry of Health, Labor and Welfare in Japan, which consists of nine chapters of strategy for the promotion of Basic Act on Hepatitis Measures

Japan has a national plan for addressing viral hepatitis called, Basic Act on Hepatitis Measures, established in 2009 (Act No. 97 of the year 2009).^3^ In the year 2011, basic guidelines for promotion of control measures for hepatitis was issued by the government, comprising nine principles of measures, in order to promote measures to prevent hepatitis B and C (Fig. 1).^4^ There are set targets and the government has allocated funding for the plan. The Ministry of Health and Labour and Welfare in Japan has appointed working groups for viral hepatitis, The Council for Promotion of Hepatitis Measures, including epidemiologists and clinical researchers. Based on the Basic Act on Hepatitis Measures, every prefecture and government has selected the linked regional core centers for the treatment of liver disease (hereafter referred as regional core centers) along with achieving cooperation of specialized medical institutions, so that there is no bias by region, and for it to be equally improved. Consultation Center for Liver diseases has been installed in all of regional core centers corresponding to the consultation from the patient and their family. The Hepatitis Information Center was established in The National Center for Global Health and Medicine, The Research Center for Hepatitis and Immunology, in 2008. Some of the roles of the Hepatitis Information Center are support for the sharing of medical information between regional core centers, the training of medical personnel, and the provision and dissemination of up-to-date information regarding hepatitis. Along with the update of Basic guidelines for promotion of control measures for hepatitis in 2016, The Hepatitis Information Center has been assigned to assume more responsibility to promote the linkage to the care of hepatitis patients, by active participation in the collaboration among regional core centers, specialized medical institutions, and central/local governments.

## SCREENING, DIAGNOSIS, AND MANAGEMENT OF VIRAL HEPATITIS IN JAPAN

Testing for viral hepatitis is an initial step of the linkage to care for patients with hepatitis B or C. In Japan, pregnant women are routinely screened for HBsAg since 1986, the cost of which is included in the health insurance program. From January 1986, hepatitis B vaccination with HB immunoglobulin to babies born to HBV carrier mothers has been available free of charge. And since April 1995, it has been available for insurance adaptation. Such selective HB vaccinations to high-risk babies have attained great success in the prevention of mother-to-child transmission in Japan. High-risk populations are not eligible for free vaccination, but some health care workers receive screening and vaccinations depending on the employer. From October 2016, birth dose hepatitis B vaccination to newborn babies has become available free of charge.

In Japan, national and local governments share screening costs for testing hepatitis B and C in those residents who are over 40 years old basically. Thus, out-of-pocket expenses from examinees are nil or reduced to the minimum. From 2001 to 2014, approximately 17 million persons have taken hepatitis virus test in this country. Test results for hepatitis B and hepatitis C are notified, and positives are encouraged to visit medical institutions and get registration to the follow-up system in some prefectures.

**Fig. 2:**
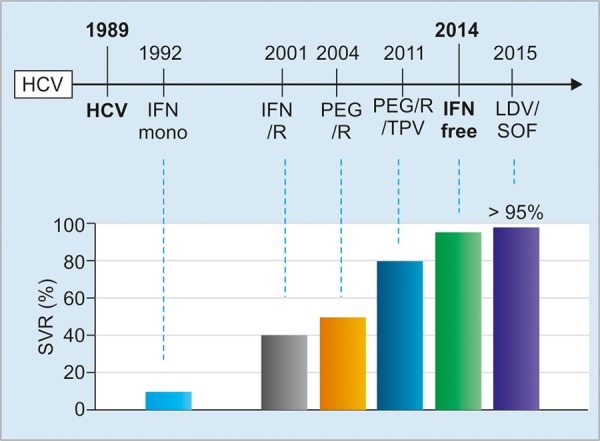
Milestones of anti-HCV treatment and corresponding sustained viral response in Japan. PEG: Pegylated interferon; R: Ribavirin; TPV: Telaprevir; LDV: Ledipasvir; SOF: Sofosbuvir

The treatment for viral hepatitis could be provided to all parts of Japan. The Japan Society of Hepatology has developed guidelines for the treatment for hepatitis B and C,^5^ and the government has research groups that provide guidelines as well. Such guidelines have been updated in pace with the registration of novel DAAs. There are no

barriers to prescribing antiviral drugs or DAAs to patients with hepatitis C with public health insurance. In addition, to include the treatment costs of chronic hepatitis B or C, drug prices for nucleotide analogs, interferon (IFN)-based treatment or IFN-free DAAs, and examination expenses should be covered by special programs for viral hepatitis. The national and local governments altogether cover the amount in excess of 100 to 200 USD (10 or 2,000 yen) of the cost of treatment (depending on the amount of tax payment). As for the eligibility of taking such coverage programs, the patients have to submit an application to the prefecture office with the recommendation from the designated hepatologist or gastroenterologist. As of December 2016, four IFN-free regimens of DAAs have been available for patients with genotype 1 HCV infection, and two regimens for those with genotype 2 infection. The sustained viral response (SVR) rate attained by anti-HCV treatment has been dramatically improved. For patients with HCV genotype 1 infection, SVR attainable by a DAA regimen is more than 95% in clinical practice (Fig. 2).

## NONALCOHOLIC FATTY LIVER DISEASE IN JAPAN

Nonalcoholic fatty liver disease (NAFLD) is recognized as the most common cause of chronic liver disease, the proportion of which in the general population is estimated to be 15 to 30% in the world. The NAFLD is a clinical entity representing of metabolic syndrome, often complicating with cardiovascular disease, obesity, diabetics, or dyslipidemia. Unless metabolic dysfunction is corrected, NAFLD is deemed as a progressive disease as the same as chronic viral hepatitis, shifting from simple steatosis, steatohepatitis, liver cirrhosis, to HCC. Nonalcoholic ste-atohepatitis (NASH), consisting of 10 to 20% of NAFLD patients, is considered as a serious form of NAFLD, because the risks of developing end-stage liver cirrhosis and HCC are increased compared with that from simple steatosis. In Japan, the proportion of liver cancer with nonviral etiology (non-B, non-C) has been increasing significantly, which has become one of the major health issues in Japan.^6^

## INTERLEUKIN-34 AS A FIBROSIS BIOMARKER FOR NAFLD PATIENTS

From the clinical point of view, the degree of fibrosis should be one of the critical features that dictate the prognosis of patients with NAFLD. Therefore, evaluating fibrosis is crucial for the management of patients with NAFLD/ NASH, especially for those at risk of HCC. Liver biopsy still remains the gold standard for evaluating the degree of hepatic necroinflammation and fibrosis in patients with chronic liver disease. However, such invasive procedures have substantial limitations; fatal complications are sometimes unavoidable, such as bleeding or biliary tract infection. Furthermore, repetitive biopsy for sequential assessment of the disease could not be possible in real clinical practice. Thus, several noninvasive investigations have been developed to establish the diagnosis and also to evaluate treatment response. For this purpose, risk scores, biomarker panels, and ultrasonography modalities have been used in the clinic to identify patients at risk of NASH without recourse to liver biopsy.

Clinical and basic researchers have been focusing on the mechanisms of progression from steatosis to steato-hepatitis/NASH. In the machineries of fibrogenesis in NASH, activation of macrophages and hepatic stellate cells (HSCs) and their interaction could play essential roles. Therefore, we hypothesized that biological factors relating to these cells could be indicators of activation of macrophages and HSCs, and serve as fibrosis-related markers in patients with NAFLD/NASH. Interleukin-34 (IL-34) is a ligand for colony-stimulating factor-1 receptor (CSF-1R). The IL-34 binds to CSF-1R and promotes differentiation, proliferation, and survival of monocytes and macrophages, the function of which is the same as the other CSF-1R ligand, macrophage colony-stimulating factor (M-CSF). We, thus, aimed to explore the feasibility of IL-34 as a fibrosis marker in patients with NAFLD.

We enrolled 197 liver biopsy-proven NAFLD patients with various fibrosis stages. We comprehensively evaluated the serum levels of macrophage-related markers (IL-34, M-CSF, soluble CD163), 40 cytokines/chemokines, hyaluronic acid, type IV collagen 7s, and clinically approved fibrosis scores, such as aspartate aminotransferase to platelet ratio index (APRI), fibrosis 4 (FIB-4) index, NAFLD fibrosis score. We performed uni- and multivariate analyses, receiver operating characteristic, and multivariate regression analyses for the assessment of diagnostic performance of various markers/indices. In order to clarify the source of IL-34, we performed immunohistochemical and immunofluorescence staining of frozen liver specimens obtained from NAFLD patients.

**Figs 3A to D:**
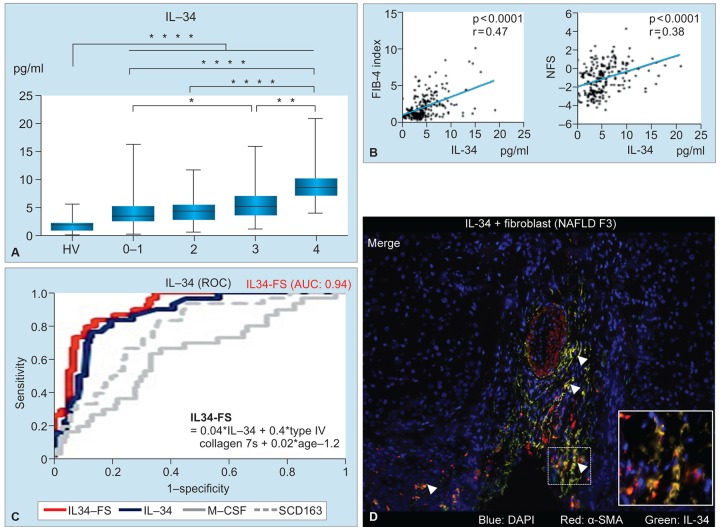
The IL-34 is a biomarker of liver fibrosis in NAFLD. In this study, 197 liver biopsy-proven NAFLD patients with various fibrosis stages were enrolled. (A) IL-34 significantly increased with the progression of fibrosis; (B) positive correlations were observed between IL-34 and FIB-4 or NAFLD fibrosis score (NFS); (C) the AUC of IL-34-FS was 0.94, which was superior to IL-34 alone, M-CSF, or sCD163; (D) liver specimen from patients with NAFLD (fibrosis stage 3) was stained for IL-34 and a-SMA. Immunostaining shows that almost all of the IL-34-positive cells in the liver tissue were fibroblasts (merged a-SMA-positive cells). *Arrowheads:* IL-34 and a-SMA double-positive cells (merged yellow cells)

The IL-34 significantly increased with the progression of fibrosis and was an independent marker for liver cirrhosis (odds ratio = 1.233, p = 0.006) (Fig. 3A). For the diagnosis of liver cirrhosis, the area under the curve (AUC) and sensitivity and specificity of IL-34 (0.87, 83.3, and 80.2% respectively) were superior to or comparable with the other serum biomarkers and fibrosis indexes. Positive correlations were observed between IL-34 and other fibrosis markers, such as FIB-4 or NAFLD activity score (Fig. 3B). The combination of serum IL-34, type IV collagen 7s, and ages, which are independent factors of liver fibrosis [IL-34-based fibrosis score (IL-34-FS) = 0.0387 × IL-34 (pg/mL) + 0.3623 × type IV collagen 7s (ng/mL) + 0.0184 × age (year) - 1.1850] was a practical tool for predicting the stages of fibrosis in NAFLD patients. The AUC, sensitivity, and specificity of IL-34-FS were 0.86, 75.2, and 85.0% (significant fibrosis), 0.88, 81.7, and 79.4% (advanced fibrosis), and 0.94, 83.3, and 85.6% (liver cirrhosis) respectively (Fig. 3C). Immunostaining revealed that almost all of the IL-34-positive cells in the liver tissue were fibroblasts (a-smooth muscle actin [SMA]-positive cells) (Fig. 3D). We demonstrated that IL-34 is feasible for evaluating the degree of fibrosis in NAFLD patients; the overall predictive accuracy is superior to the other biomarkers.^7^

## PERSPECTIVES

Synthetic compounds that specifically suppress HBV or HCV replication are now used in clinics. They are quite promising as an alternative approach for hard-to-treat chronic hepatitis patients, such as those infected with drug-resistant HBV or HCV strain, and compensated or decom-pensated liver cirrhosis. In addition to inhibitory effect on viral replication, such compounds are able to restore immunity either indirectly by reducing viral burden or directly by immune modulation. Therefore, extensive and long-term follow-up studies are needed to elucidate if viral eradication with DAAs could actually reduce the incidence of HCC afterward. Noninvasive biomarkers of liver fibrosis, such as IL-34 identified by us, is of clinical importance for the follow-up of patients who attained SVR.

Most of the people living in middle- or lower-income countries hardly get access to costly brand-new drugs. In addition, newly/repetitive HCV-infected patients are on the rise because of the expansion of injection drug users due to social irritability. In order to solve such socioeconomic and health care problems, the development of protective/therapeutic vaccines could be one of the remedies. Several beneficial advantages have prevailed in Japanese health care systems for patients with viral liver disease compared with those in countries in the Western Pacific region. In our personal opinion, Japan should take a lead in helping the implementation of practical hepatitis action plan adjusted for each country when in need.
